# Equine trypanosomiasis, a systematic review: Disease management

**DOI:** 10.1002/evj.70136

**Published:** 2025-12-22

**Authors:** Alexandra G. Raftery, Lauren Gummery, Karelhia Garcia, Dinesh Mohite, Paul Capewell, David Sutton

**Affiliations:** ^1^ School of Biodiversity, One Health and Veterinary Medicine, College of Medical, Veterinary and Life Sciences University of Glasgow Glasgow UK; ^2^ Three Counties Equine Hospital, Stratford Bridge, Ripple, Tewkesbury, Gloucestershire UK; ^3^ Faculty of Veterinary Medicine, Veterinary Medical Sciences University of Calgary Calgary Alberta Canada; ^4^ Federation of Indian Animal Protection Organisations New Delhi India; ^5^ School of Molecular Biosciences, College of Medical, Veterinary and Life Sciences University of Glasgow Glasgow UK

**Keywords:** donkey, Dourine, horse, Nagana, Surra, trypanosomiasis

## Abstract

**Background:**

Equine trypanosomiasis is a neglected protozoal disease.

**Objectives:**

To answer the study question: In equines what are the effects of disease management of trypanosomiasis on disease severity (individual level) and disease prevalence (population level) compared to no intervention?

**Study Design:**

Systematic review.

**Methods:**

Studies were identified that described management of naturally occurring equine trypanosomiasis in any country following ‘Preferred Reporting Items for Systematic Reviews and Meta‐analyses’ using eight international databases (1980–2022). Risk of bias was assessed using ROBINS‐I. Data synthesis was descriptive.

**Results:**

Thirty studies were included (9 case reports, 5 case series, 15 cohorts, 1 randomised non‐inferiority trial). Risk of bias was ‘serious’ (22/30), ‘moderate’ (7/30), ‘low’ (1/30). Heterogeneity was high. Disease severity (individual): *Trypanosoma evansi*: all evaluated trypanocides were effective in blood parasitaemia clearance (weak evidence). Clinical relapses were common (*n* = 60/241 equines treated; 25%) (strong evidence). Efficacy was poor once neurological signs were present (*n* = 12/19 equines; 63% mortality) (strong evidence). *Trypanosoma equiperdum*: a combination protocol could be curative before CNS invasion (weak evidence). *Tsetse transmitted trypanosomiasis*: Treatment of haemolymphatic disease with isometamidium or diminazene resulted in a positive clinical response (strong evidence). New/recrudescing infections were common in some regions (strong evidence). *Trypanosoma vivax*: treatment with high‐dose diminazene had a poor clinical outcome (weak evidence). Disease prevalence (population): a multifaceted control programme was effective in reducing disease prevalence (weak evidence). Early (<2 days post‐infection) treatment was more effective (weak evidence). Reported side effects were uncommon (*n* = 70/7888 equines; 1%) (strong evidence). Isometamidium chloride (0.5 mg/kg i.v.) can cause a shock response (13%; range 10–14; *n* = 14/105) (strong evidence).

**Main Limitations:**

Publication bias, heterogeneity, descriptive data.

**Conclusions:**

Short‐term trypanocide response for haemolymphatic disease was positive but optimisation of treatment protocols is required to reduce relapse and combat neurotrypanosomiasis. Reliance on trypanocidal treatment alone is common. Side effects are rare but can be severe.

## 1 INTRODUCTION

Trypanosomiasis presents as a haemolymphatic and/or neuropathological disease. The disease results in three clinical syndromes (Nagana, Surra and Dourine). These syndromes are clinically defined by their mode of transmission which also defines their geographical distribution. Greater background detail can be found in a related evidence review.^1^

Equine trypanosomiasis is a significant contributor to global equine morbidity and mortality.^2^ Previous work on clinical treatment efficacy identified that disease management guidelines were incomplete and data relating to the efficacy of interventions were limited.^3^, ^4^ Recommended disease management is dependent on the vector and/or mode of transmission of relevant *Trypanosoma* sp.; tsetse flies for Nagana (*Trypanosoma brucei*, *Trypanosoma congolense*, *Trypanosoma vivax*); biting flies for Surra (*Trypanosoma evansi*) and non‐tsetse transmitted *T. vivax* and venereal transmission for Dourine (*Trypanosoma equiperdum*). Current disease management options include trypanocide medications (such as isometamidium, diminazene or quinapyramine),^5^ vector control (including tsetse traps, topical insecticide and clearance of vector habitat), management interventions (such as stabling at‐risk periods with insecticide‐treated mesh netting, separating from cattle or other relevant reservoir species, euthanasia of chronically infected *Trypanosoma brucei* spp. equines) and holistic approaches (such as optimising the nutrition plane).^3^ Evidence relating to the efficacy of these interventions is limited in equines. This is further complicated by the variation in reported diagnostics used to identify and monitor infections and their associated differences in sensitivity and specificity. Diagnostics range from the visualisation of parasites by microscopic evaluation of blood film or buffy coat,^6^ isolation of parasite DNA through molecular testing of blood such as PCR^7^ or confirmation of exposure to parasites by serological testing of blood such as ELISA or CFT.^8^

We hypothesised that objective analysis would help strengthen understanding of current approaches to disease management but that factors relating to study quality and gaps in available knowledge would lead to challenges in having high confidence in conclusions. The aim of this study was therefore to consolidate existing knowledge and identify areas where future challenge‐led research is required. To assimilate the current knowledge and understanding of equine trypanosomiasis, a systematic review of peer‐reviewed and grey literature (scientific data not published through academic channels but presented within documents such as policy documents, theses and dissertations; included to reduce publication bias) was performed following the Preferred Reporting Items for Systematic Reviews and Meta‐analyses (PRISMA) guidelines^9^ and Synthesis Without Meta‐analysis (SWiM) in systematic reviews reporting guidelines.^10^

The objective of this study was to answer the following study question: In equines (*population*) what are the effects of disease management of trypanosomiasis (*intervention*) on disease severity (individual level) (*outcome 1*) and disease prevalence (population level) (*outcome 2*) compared to no intervention (*comparison*)?

## 2 METHODS

**FIGURE 1:**
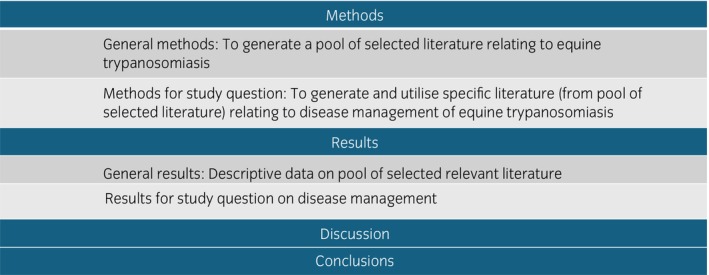
Diagram illustrating the methodological approach and structure of this systematic review of the disease management of equine trypanosomiasis. The study question on disease management utilises specific methodology that is applied to a pool of selected relevant literature to generate specific literature used to extract data to answer the question.

The detailed study protocol is available (Data S1) following PRISMA^9^ and SWiM guidelines.^10^ A pool of selected, relevant literature was generated and then specific methodology was applied to this to produce a pool of selected and specific literature which focused on disease management. For clarity the methodological approach and structure of this manuscript are outlined in Figure 1.

### 2.1 Amendments

Post‐hoc amendments to the study protocol (Data S1) are evident by comparison with the original pre‐registered protocol (available at osf.io/r6fsw).

### 2.2 General methods: To generate a pool of selected literature relating to equine trypanosomiasis

#### 2.2.1 Search strategy

References for this review were identified through searches of eight international databases and grey literature (search strategies detailed in study protocol; Data S1). Broad search terms were chosen to capture studies on any aspect of equine trypanosomiasis. Publication year was restricted to between January 1980 and July 2022 to improve the relevance of the data to current times whilst aiming to represent data from the maximum number of countries, as published studies were anticipated to be sporadic.

#### 2.2.2 Study selection and criteria

The abstracts and titles were downloaded and collated (.csv or .xls). The records were adapted into a standardised format to allow consolidation in Excel. This allowed searching for duplicity (performed manually) to remove items listed more than once.

Each title (Step 1—initial screening for topic relevance) and then title and abstract (Step 2—abstract review) were reviewed independently by two reviewers (AGR, LG) who were blinded to the decision of the other reviewer. The articles were selected based upon a predetermined, standardised inclusion and exclusion criteria. Where reviewers differed in opinion, a third reviewer (DGMS) had the deciding vote.

**FIGURE 2:**
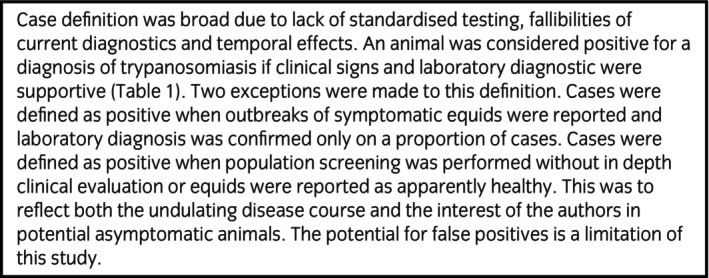
Case definition for diagnosis of equine trypanosomiasis.

Inclusion criteria for topic, title and abstract:

1. Contained original data on horse, donkey or mule *Trypanosoma* spp. infection from any country in the world.
2. Data complied with case definition (Figure 2 and Table S1, S3). Case definition was agreed by AGR, LG and DGMS.
3. All types of study design including case reports were retained to try and obtain the greatest geographical coverage of information since data were anticipated to be sparse/absent in some areas. It was anticipated that the level of evidence would be generally low since randomised controlled trials are rare in veterinary medicine and therefore all types of study design were maintained initially to gain the benefits of repeated observations.
4. Abstract written in English, French, Spanish or Portuguese due to skillset available to the authors.

Exclusion criteria for topic, title and abstract:

1. Studies reporting experimental infections.
2. Studies with insufficient information to assess case definition.
3. Studies where full text was not available.
4. Non‐systematic review articles, book chapters, newspaper articles and other documents that did not contain original data.
5. Grey literature with peer reviewed manuscripts containing the same data.

#### 2.2.3 Full text review

Articles meeting these inclusion criteria based on topic, title and abstract then underwent a review of the full text. This included particular attention that the case definition (Figure 2 and Table S1) was still fulfilled when the full text had been reviewed. The included articles were collated into Zotero^11^ reference manager to facilitate the review process. Articles requiring full text review in French, Spanish or Portuguese were translated. Articles were excluded after full text review based on the following exclusion criteria. This review was performed by AGR and LG with DGMS as a deciding vote for differing opinions.

Exclusion criteria for full text:

1. Study did not reach criteria (inclusion/exclusion) outlined in title and abstract review.

#### 2.2.4 Data extraction and synthesis

A flow chart demonstrating the number of included studies and points of exclusion was made using the PRISMA template^12^ as the study was performed (Figure [Link], [Link]). General information was extracted for each study including study design, publication year, country studied, country affiliation of primary author and sources of funding (specific details within Study protocol (Data S1), outline database pre‐registration on OSF (osf.io/ky8th/) and Tables [Link], [Link] Results). For this study question on disease management topic inclusion and exclusion criteria were generated (Section 2.3; study protocol, Data S1) to apply to this pool of relevant collated literature. Initial data extraction was performed by AGR into a standard form (Tables S2, S4), studies included within the specific study question were later re‐reviewed for more detailed descriptive data.

### 2.3 Methods for study question: To generate and utilise specific literature relating to disease management of equine trypanosomiasis

#### 2.3.1 Extraction of specific data and data analysis

The study question was devised following discussions amongst the authors about how to extract information that would be the most useful to assimilate practically to achieve the study's objective to assess the disease management of equine trypanosomiasis. This aimed to answer the question:

‘In equines what are the effects of trypanosomiasis disease management strategies on disease severity (individual level) and disease prevalence (population level) compared to no intervention?’

Studies were included that evaluated any trypanosomiasis disease management strategy (pharmacological, vector control or managemental) by reporting the number of equines positive for trypanosomiasis before and after an intervention. This inclusion criteria was applied to the pool of selected literature generated by the general methods (Section 2.3).

Based on the author's prior knowledge, a predominance of exploratory rather than confirmatory studies was expected. Thus, data compilation was planned to be predominantly descriptive through visualisation and consolidation.

#### 2.3.2 Critical assessment of methodological quality

Data quality was anticipated to be low; therefore, qualitative and quantitative information was extracted. Critical assessment of methodological quality (including risk of bias) was performed at the individual manuscript and the outcome level. Risk of bias was assessed using ROBINS‐I^13^ and visualised using the *robvis* tool^14^ tool. Heterogeneity was not formally evaluated but inferred due to the mix of study design, treatments, geography and species examined. Publication bias was assessed qualitatively. The level of confidence in individual studies was assessed using GRADE^8^, ^15^ to rate certainty as very low, low, moderate or high. Statements on outcome variables for the study question were described using GRADE^15^ to rate recommendations as strong or weak evidence, in favour of or against. Certainty was rated down for risk of bias, imprecision, inconsistency, indirectness and publication bias and certainty was rated up for a large magnitude of effect, dose–response gradient and residual confounding factors that would increase effect.

#### 2.3.3 Descriptive data extracted from included studies

Data were extracted pertaining to the number of equines in each group, the diagnostic method used, intervention made and time frame for repeat evaluation (extracted data in Tables [Link], [Link]). The outcome variables were disease severity (individual clinical improvement) and disease prevalence/incidence after the intervention. Data were also extracted pertaining to documented side effects for pharmacological interventions. Descriptive statistics of included studies were summarised in table format focused on the comparison of outcome variables.

## 3 RESULTS

### 3.1 General results

#### 3.1.1 Study selection and characteristics

The complete extracted data can be accessed (Tables S2, S4). A PRISMA flow diagram illustrates the process of selection of eligible articles for study inclusion (Figures [Link], [Link]). Descriptive data on the retrieved manuscripts are outlined in a related manuscript ‘Equine Trypanosomiasis: a systematic review of prevalence, morbidity and mortality’.^1^

### 3.2 Results for study question on disease management

#### 3.2.1 Search outcome

Quantity of evidence: Thirty of the 205 papers (15%) met the inclusion criteria for this question.

**FIGURE 3:**
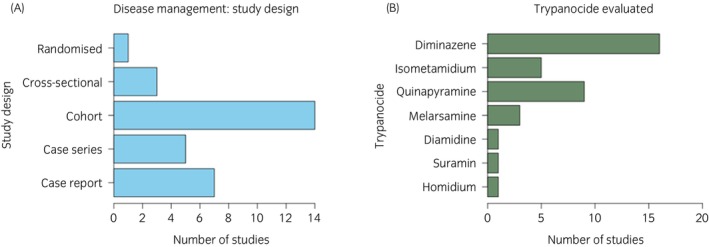
Descriptive data of equine trypanosomiasis disease management studies. (A) Study design of included studies. (B) Trypanocides evaluated in studies.

Study design: There were seven case reports, five case series, 14 cohort studies, three cross‐sectional studies and one randomised non‐inferiority trial (Figure 3).

The manuscripts represented 14 countries across Africa (*n* = 7), Asia (*n* = 18), Central and South America (*n* = 4) and Europe (*n* = 1). All manuscripts evaluated included at least one pharmacological intervention. Four manuscripts evaluated a multifaceted disease control programme including vector control and management interventions. There were no manuscripts with untreated controls.

The risk of bias (visualised in Tables [Link], [Link]) was assessed using ROBIN‐I overall as ‘serious’ in 22/30, ‘moderate’ in 7/30 and ‘low’ in 1/30 studies.

Qualitatively heterogeneity was high originating from clinical (*Trypanosoma* sp., trypanocide, dose of trypanocide, equine species), methodological (sample population, inclusion criteria, outcome variable, diagnostic tests) and statistical heterogeneity (assessment of reported intervention efficacy).

#### 3.2.2 Disease severity (individual)

All included studies evaluated the use of one or more trypanocide treatments on individual outcomes with information on dose and follow‐up available. From the 26 relevant manuscripts, there was one randomised non‐inferiority trial, five case series, 12 cohort studies and eight case reports. The studies reported on diminazene aceturate (*n* = 16), isometamidium chloride (*n* = 5), quinapyramine sulphate/quinapyramine chloride (*n* = 9), melarsomine dihydrochloride (*n* = 3), diamidine hydrochloride (*n* = 1), suramin (*n* = 1) and homidium chloride (*n* = 1) (Figure 3). No studies were available that evaluated the impact of vector control or management interventions on individual outcomes. The outcomes are summarised by *Trypanosoma* sp. (Tables 1, 2, 3, 4).

**TABLE 1:** Disease severity: evaluation of trypanocidal efficacy against *Trypanosoma evansi* in naturally occurring equine trypanosomiasis.

Trypanocide	Study design	Baseline	Post‐intervention
Diminazene aceturate	5 case reports^16^, ^17^, ^18^, ^19^, ^20^ 3 case series^21^, ^22^, ^23^ 2 cohort studies^24^, ^25^	**53 equines** (9 mules, 2 donkeys) 3.5 mg/kg i.m. (*n* = 42) second treatment (*n* = 24) (7 mg/kg) 4.05–10 mg/kg i.m. (*n* = 11); repeated doses of 4.05 mg/kg q3 days (*n* = 6).	**Mortality post‐treatment:** 18/53 (33%) died (survival follow‐up 7 days‐6 months). Neurological signs at presentation 9/15 (60%) died **Morbidity post‐treatment:** Initial post‐treatment clearance in parasitaemia in all where checked. In survivors, post‐treatment clinical signs common 24/35 (69%)
Isometamidium chloride	1 case series^26^	**6 equines** 0.5 mg/kg i.m.; 2% solution	Day 7 6/6 (100%) negative parasitaemia, remained anaemic
Quinapyramine sulphate (QS)/quinapyramine hydrochloride (QC)	1 case report^27^ 2 case series^26^, ^28^ 5 cohort^24^, ^29^, ^30^, ^31^, ^32^	**79 equines (3 donkeys, 4 mules)** Quinapyramine sulphate 2.5‐3 mg/kg, quinapyramine chloride 1.7‐2 mg/kg s.c. (QS only *n* = 14)	**Diagnostics:** Negative parasitaemia reported in 50/50 (100%) at first evaluation (day 4, 7 or 70) post‐treatment given QS/QC 2.5–3/1.7‐2 mg/kg s.c.^26^, ^27^, ^29^, ^30^, ^32^ (*n* = 6 no dose). Antibody positive 6–13 months follow‐up (5/37; 14%)^29^, ^32^ **Clinical response:** Remained anaemic 7 days post‐treatment (*n* = 6)^26^ 8/58 (14%) relapsed: 4/58 (7%) developed neurological signs (27–151 days) 7/58 (12%) died, 1 day to 5 months post‐treatment (follow‐up 4–5 months) 3/58 (5%) had neurological signs at time of treatment; 2/3 died, 1/3 alive at 4 months^24^, ^28^, ^31^, ^32^
Melarsomine dihydrochloride	2 cohort studies^24^, ^33^	**16 equines (7 donkeys)** 0.25–0.5 mg/kg i.m.	4/6 (71%) died (plus one with neurological signs prior to treatment)^24^ Post‐treatment negative parasitaemia but positive serology (*n* = 9; 2 months)^33^

*Note*: There was variation in the assessment of outcome with infection status including parasitaemia assessed using different techniques (diagnostics utilised for each study recorded in Table S3; assessment of parasitaemia was by evaluation by microscopy of Giemsa stained blood smear, evaluation of wet blood smear or use of the micro‐haematocrit centrifugation technique to evaluate the buffy coat).

**TABLE 2:** Disease severity: evaluation of trypanocidal efficacy against *Trypanosoma equiperdum* in naturally occurring equine trypanosomiasis.

Trypanocide	Study design	Post‐intervention
Diminazene aceturate (3 mg/kg s.c. q24 hours for 3 days) then quinapyramine sulphate 3 mg/kg s.c. twice in 7 days	1 case report^34^	Haemolymphatic and reproductive pathology. Mild peripheral neuropathy prior to treatment (unilateral facial nerve paralysis) Clinical signs resolved by 2 months post‐treatment, negative on all diagnostics. Remained clinically and diagnostically negative including serology for 2.5‐year follow‐up period

**TABLE 3:** Disease severity: evaluation of trypanocidal efficacy against *Trypanosoma vivax* (non‐tsetse transmitted) in naturally occurring equine trypanosomiasis.

Trypanocide	Study design	Post‐intervention
Diminazene aceturate (7 mg/kg i.m. q7 days ×3)	1 case series^35^ 6 horses	Initial positive response to treatment. Clinical signs recurred 3 times (4 months onwards). Treatment repeated each time. 3/6 (50%) died/euthanised due to persistent clinical signs. 3/6 (50%) survived with resolution of clinical signs

**TABLE 4:** Disease severity: evaluation of trypanocidal efficacy against tsetse‐transmitted trypanosomiasis in naturally occurring equine trypanosomiasis.

Trypanocide	Study design	Baseline	Post‐intervention
Diminazene aceturate	1 cohort study^36^	**16 horses** 3.5–7 mg/kg i.m.	**Descriptive data (4 weeks post‐treatment)** 14/16 (88%) had a positive clinical response, return to normal PCV and negative parasitological tests/Antigen ELISA; 1 required repeat treatment (6 mg/kg i.m.); 1/16 (6%) died (*Trypanosoma brucei*, *Trypanosoma congolense*). No neurological signs noted.
	1 randomised, open label, non‐inferiority trial^4^	**51 equines** (35 horses, 16 donkeys) 3.5 mg/kg i.m., 5% solution, in 2 aliquots T0: All met clinical inclusion criteria, confirmed PCR positive ≥1 *Trypanosoma* sp., no neurological signs.	**Analytical data (2 weeks post‐treatment)** **Treatment response:** Improved demeanour, body condition score and reduction in rectal temperature. Significant increase in PCV (5% [1–10]). Reduction in PCR positivity (*Trypanosoma vivax* 45 vs. 0%; *T. congolense* 68 vs. 13%; *T. brucei* 22 vs. 2%) **Comparative effect by PCR status** **By *Trypanosoma* sp.:** *T. vivax*: non‐inferior to isometamidium; *T. congolense*: inferior to isometamidium; *T. brucei*: non‐inferiority to isometamidium not demonstrated, not excluded **By whole animal PCR status:** The point estimate favoured isometamidium, non‐inferiority of diminazene was not demonstrated **New/reinfection:** 1/85 (1%) equines (*T. brucei*)
Isometamidium hydrochloride	1 cohort study^37^	**40 donkeys** 1 mg/kg i.m.	**Descriptive data:** Monitored PCV and parasitaemia every 2 weeks. New/reinfections at 1 month 8/40 (20%); at 3 months 28/40 (70%) new/reinfections. Seventy percent *T. congolense*
	1 randomised, open label, non‐inferiority trial^4^	**53 equines** (18 horses, 35 donkeys) 0.5 mg/kg i.v. 0.5% solution	**Analytical data (2 weeks post‐treatment)** **Treatment response:** Improved demeanour, body condition score and reduction in rectal temperature. Significant increase in PCV (6% [2–10]). Reduction in PCR positivity (*T. vivax* 58 vs. 4%; *T. congolense* 64 vs. 0%; *T. brucei* 28 vs. 0%) **Comparative effect by PCR status** **By individual *Trypanosoma* sp.:** *T. vivax*: Isometamidium superior to melarsomine. Diminazene non‐inferior to isometamidium; *T. congolense*: Isometamidium superior to melarsomine and diminazene aceturate; *T. brucei*: Isometamidium chloride superior to melarsomine dihydrochloride. Diminazene non‐inferiority not demonstrated but not excluded **By whole animal PCR status:** Neither diminazene aceturate nor melarsomine dihydrochloride could demonstrate non‐inferiority to isometamidium **New/reinfection: 6**/85 (7%) treated equines (*T. congolense n* = 3; *T. vivax n* = 1; T. *brucei* sp. *n* = 2)
Melarsomine dihydrochloride	1 randomised, open label, non‐inferiority trial^4^	**58 equines** (21 horse, 37 donkeys) 0.25 mg/kg i.v. (0.5% solution)	**Analytical data (2 weeks post‐treatment)** **Treatment response:** Improved demeanour, body condition score and reduction in rectal temperature. Significant increase in PCV (3% [0–6]). Reduction in PCR positivity (*T. vivax* 45 vs. 27%; *T. congolense* 71 vs. 57%; *T. brucei* 21 vs. 8%). **Comparative effect (to isometamidium) upon PCR status** **By individual *Trypanosoma* sp.:** *T. vivax*: Inferior to isometamidium. Effect comparable to placebo not excluded; *T. congolense*: Inferior to isometamidium, comparable to placebo; *T. brucei*: Inferior to isometamidium. Effect comparable to placebo not excluded **By whole animal PCR status:** Inferior to isometamidium, effect comparable to placebo **New/reinfection**: 10/87 (12%) treated equines (*T. congolense n* = 5; *T. vivax n* = 3; T. *brucei* sp. *n* = 1)

A multitude of supportive and complementary therapies was utilised. Of note, six out of seven case reports documented the use of antibiotics as a first‐line symptomatic treatment prior to administration of a trypanocide. There were six manuscripts where antihistamines were administered prior to trypanocide treatments.

##### Disease severity: *T. evansi* treatment

The level and quality of evidence available to assess trypanocidal treatment efficacy for *T. evansi* is low (Tables 1 and S3, S5). There was *weak evidence in favour* of each evaluated trypanocide being capable of effective post‐treatment clearance of parasitaemia (variable diagnostics [specific details in Table S5: Table S3] and time frame; no controls). For each evaluated trypanocide there were equines that did respond positively (variable follow‐up). Mortality was high (*n* = 36/154; 24%). The data are too heterogeneous to compare outcomes quantitatively. Trypanocidal treatment (diminazene aceturate, melarsomine dihydrochloride or quinapyramine sulphate/chloride) was generally ineffective when neurological signs were present (*n* = 12/19 equines; 63% mortality; variable follow‐up).

##### Disease severity: *T. equiperdum* treatment

There is *weak evidence in favour* of exploring curative treatment in early *T. equiperdum* infections/exposure. The combination protocol (diminazene aceturate 3 mg/kg s.c. q24 hours for 3 days; quinapyramine sulphate 3 mg/kg s.c. twice in 7 days) (Table 2) should be accompanied by clinical staging to inform prognosis and cessation of breeding activity pending long‐term clinical and diagnostic follow‐up.

##### Disease severity: Tsetse transmitted trypanosomiasis treatment

Extracted data are summarised in Table 4. There is *strong evidence in favour* of a short‐term positive clinical and clinicopathological response in equines (donkeys and horses) with haemolymphatic disease due to tsetse transmitted trypanosomiasis (*T. congolense*, *T. vivax* and/or *T. brucei* sp.) following treatment with isometamium chloride (0.5 mg/kg i.v. 0.5% solution) or diminazene aceturate (3.5 mg/kg i.m., 5% solution, in 2 aliquots). This is supported by the concurrent reduction in *Trypanosoma* sp. PCR positivity. Where there is a mixed *Trypanosoma* sp. challenge isometamidium chloride is the preferred option (*strong evidence*, *in favour*) but melarsomine dihydrochloride (0.25 mg/kg i.v.; 0.5% solution) is not an appropriate treatment (*strong evidence*, *against*). New/recrudescing infections present an important challenge in some regions (*strong evidence*, *in favour*). Quinapyramine sulphate/chloride has not been evaluated with the outcome variable as disease severity. For equines, once neurological signs are evident (*T. brucei* sp.) there are no studies supporting the efficacy of trypanocidal intervention (*weak evidence*, *against*).

##### Disease severity: *T. vivax* treatment

Extracted data are summarised in Table 3. Treatment of *T. vivax* (non‐tsetse transmitted) with diminazene aceturate (high dose 7 mg/kg i.m. q7 ×3) did not result in a good clinical outcome (3/6 died; 50% mortality). There is *weak evidence against* pursuing this protocol.

**TABLE 5:** Disease prevalence: evaluation of key features of population‐level intervention studies for the management of equine trypanosomiasis.

Study	*Trypanosoma* sp.	Country	Study design	Intervention	Baseline	Post‐intervention
Waheed et al.^38^; Waheed et al.,^39^ Gondal and Ahmad^40^	*Trypanosoma evansi*	Pakistan	Analytical, longitudinal, interventional cross‐sectional	**1. Pharmacological:** Isometamidium chloride (0.5 mg/kg i.v. in 500 mL dextrose) followed by Quinapyramine sulphate/Quinapyramine chloride (10 mg/kg s/c in 10–15 mL distilled water) at later time point. Nursing care (rest, good diet), aspirin and multivitamin. Follow‐up treatment. Used as regular treatment protocol **2. Vector control:** Improve drainage (↓fly breeding), kerosene oil on stagnant water, spray stable walls, spray animal q3 weeks (0.5% methoxychior), cover wounds, use disposable syringes, isolate cases, surround with smoke ring (prevent fly access), euthanise when prognosis poor, bury carcass	Region level, clinic‐based intervention (73,497 equines—32,434 horses, 29,766 donkeys, 11,297 mules) Prevalence in local clinics was increasing (21–38%)	Protocol introduced: prevalence ↓ 3.96% over 13 years (clinical examination, wet and Giemsa‐stained blood smear)
Faye et al.^6^	Tsetse transmitted trypanosomiasis (*Trypanosoma vivax*, *Trypanosoma congolense*, *Trypanosoma brucei*)	Gambia	Descriptive, cohort, longitudinal	**1. Pharmacological:** Single dose of diminazene aceturate 3.5 mg/kg i.m. (7 mg/kg for *T. brucei*) for 13 months if infected. Equids tested monthly (buffy coat examination). Village‐level intervention (78 equines—11 horses; 67 donkeys). PCR used for monitoring	No baseline incidence details	Overall average monthly incidence in untreated animals 11% versus 7% in treated group. Estimated 18 days protection
Auty et al.^41^	Tsetse transmitted trypanomiasis (*T. vivax*, *T. congolense*, *T. brucei*)	Tanzania	Descriptive, cohort, longitudinal	**1. Pharmacological:** Quinapyramine sulphate (2.5 mg/kg) and chloride (1.7 mg/kg) s/c for treatment; isometamidium chloride 0.5 mg/kg i.m or quinapyramine for regular prophylaxis every 3 months **2. Vector control:** acetone baited tsetse control targets treated with deltmethrin. Synthetic pyrethroid application topically on horses. Minimal stable lighting at night time to reduce insect attraction. Thick vegetation cut back around the buildings **3. Management:** Careful twice daily clinical monitoring and rapid treatment	No baseline incidence details. Premises‐level intervention (24 horses over 23 months) Diagnosis: clinical examination (temp 2× day), PCV, microscopy (buffy coat evaluation) and PCR	Incidence rate covered by prophylaxis: 0.39 cases/horse‐year at risk versus 0.98 when not covered. Incidence rate ratio 2.5 (95% CI 0.84–7.36) Ataxia, weight loss & severe anaemia (<20%): significant risk factors for death

##### Disease severity: All

Clinical relapses post‐treatment including development of neurological disease and death, were common post‐trypanocidal treatment (all) where follow‐up >1 week was available (diminazene aceturate, melarsamine dihydrochloride, quinapyramine sulphate/chloride) (*n* = 60/241 equines treated; 25% relapse).

#### 3.2.3 Disease prevalence (population)

Extracted data of the five cohort studies reporting a population level intervention are summarised in Table 5. There is *weak evidence against* trypanocidal treatment as a sole intervention. There is *weak evidence in favour* of a multifaceted disease control programme (including targeted +/− prophylactic trypanocidal use, vector control and management interventions) to reduce local incidence or prevalence of disease. The relative effect size of each aspect of the control programme is not known. There was *weak evidence in favour* of early (less than 2 days post‐infection) trypanocidal treatment being more effective.

#### 3.2.4 Side effects

The majority of reported trypanocidal use in equines was without documented immediate side effects (*n* = 7818/7888 equines; 99%) (*strong evidence in favour*) (Table 6). There is *strong evidence in favour* of isometamidium chloride (0.5 mg/kg i.v.) causing an immediate mild to severe pain and/or shock response (described as significant tachycardia, tachypnoea and cold peripheries) in 13% (range 10–14; 14/105) of treated equines (*n* = 3 studies, one descriptive), which has the potential to be fatal. There is *weak evidence against* associating diminazene aceturate (3.5 mg/kg i.m.) with immediate moderate to severe signs of anaphylaxis (single manuscript; signs recurred on repeat treatment, *n* = 8/16; no other reports in *n* = 1514 treatments). There is *weak evidence in favour* (one manuscript, *n* = 15/15 horses, detailed clinical observations) of immediate reactions (colic, sweating, restlessness) to quinapyramine sulphate/quinapyramine chloride (2.5/1.7 mg/kg s.c.). No other reports were noted (*n* = 3072 treatments).

**TABLE 6:** Side effects: summary of frequency of documented side effects following trypanocidal treatment in naturally occurring equine trypanosomiasis.

Trypanocide	Data sheet dose	Study designs	Number of treatments and dose	Side effects
Diminazene aceturate	3.5 mg/kg i.m. once 7 mg/kg i.m. once (*Trypanosoma brucei* infections) 1520/1522 (99%) in data sheet range for single/first treatment	6 case reports^16^, ^17^, ^18^, ^19^, ^20^, ^34^ 3 case series^21^, ^23^, ^35^ 6 cohorts^6^, ^24^, ^25^, ^36^, ^42^, ^43^ 1 randomised non‐inferiority trial^4^	1522 equines (>120 donkeys, 9 mules). Majority of reports single study (1214/1522; 80%)^43^ Dose: 3.5–10 mg/kg i.m. 1 report of s.c.^34^ 5 studies^21^, ^23^, ^24^, ^34^, ^35^ (54 equines): repeated dosing (3.5 mg/kg q24 hours ×3—7 mg/kg i.m. weekly ×3)	**Immediate reaction (3.5 mg/kg)** ^23^: Severe reaction (salivation, restlessness, recumbency, dyspnoea). T0: 5/8 horses, 1/8 mules. T41 (second treatment): 4/8 horses, 4/8 mules, moderate to severe (lip oedema, salivation, recumbency, dyspnoea, dysphagia); 1 horse died **Delayed reaction (3.5 mg/kg i.m.)** **Injection site reactions:** 2 studies Mild 1 and 2 weeks post‐injection (13/85, 15%^4^). Significant, oedematous (number not recorded, 49 treated)^42^ **Other temporally associated** ^4^: Diarrhoea 3/85 (4%), abortion 1/85 (1%) (similar to background level)
Isometamidium hydrochloride	0.5 mg/kg i.m., 2% solution, 1.5‐inch needle, between 2 and 3 sites. Repeat q10‐16 weeks (prophylaxis)	1 case series^26^ 4 cohort^37^, ^41^, ^42^, ^43^ 1 cross‐sectional^40^ 1 randomised non‐inferiority trial^4^	3175 equines (>87 donkeys). Majority of reports from single study.^40^ 0.5 mg/kg i.v. (*n* = 3015) In 500 mL of dextrose (*n* = 2910), 0.5% solution (*n* = 85). 0.5 mg/kg i.m. (*n* = 120) 1 mg/kg i.m. (*n* = 40)	**Immediate side effects**: 3/3 studies 0.5 mg/kg i.v. Observation (*n* = 2910 equines): ‘If overdose or too fast; restlessness, salivation, sweating, shivering, frequent defaecation can occur. Can cause death’. No quantitative data.^40^ Donkeys (12/47; 26%), horses (0/38): Mild‐ severe pain/shock response (↑↑HR, ↑↑RR; cold peripheries), resolved <1 h. No intervention. Characteristic linear sweating.^4^ Horses (2/20; 10%) reacted as if sedated, nervousness, diarrhoea for 30 mins.^42^ **Delayed side effects:** 1 study^4^ 0.5 mg/kg i.v.: Temporally associated: diarrhoea 2/85 (2%), 5/85 (6%) mild injection site reaction. Similar to background level.
Quinapyramine sulphate and chloride	Quinapyramine sulphate (QS) (2.5 mg/kg) Quinapyramine chloride (QC) (1.7 mg/kg) i.m., 83% solution (2.5 g + 30 mL sterile water), 2–3 sites. q2‐3 months (prophylaxis)	2 case reports^27^, ^34^ 2 case series^26^, ^28^ 6 cohort^24^, ^29^, ^30^, ^31^, ^32^, ^41^ 1 cross‐sectional^40^	3087 equines (>2 mules, 1 donkey). Majority of reports from single study.^40^ QS (6 mg/kg)/ QC (4 mg/kg) s.c. in 10–15 mL distilled water (*n* = 2910); QS (2.5 mg/kg); QC (1.7 mg/kg) given together s.c. (*n* = 128); QC (2 mg/kg) given together s.c. (*n* = 10); QS/QC 3–4 mg/kg s.c. (*n* = 39); QS 3 mg/kg s.c. (*n* = 15)	**Immediate side effects:** 1 study^41^ QS (2.5 mg/kg); QC (1.7 mg/kg) given together s.c.: 15/15 horses: colic, sweating, restlessness for 0.5–3 hours **Delayed effects**: 1 study^41^ 15/15 horses, localised non‐painful injection site up to several months.
Melarsomine dihydrochloride	0.25 mg/kg i.m. as 0.5% solution	2 cohort studies^24^, ^33^ 1 randomised non‐inferiority trial^4^	104 equines: Majority single study (*n* = 87)^4^ 0.25–5 mg/kg i.v. /i.m./s.c.	**Delayed effects:** 1 study^4^ Temporally associated: diarrhoea 4/87 (5%), abortion 2/87 (2%), 3/87 (3%) mild injection site reaction. Similar to background level

*Note*: No side effects were documented in a single study to homidium bromide (1 mg/kg i.m. in 90 equines^43^) (no information on efficacy available).

Abbreviations: HR, heart rate; RR, respiratory rate.

Evaluation of delayed side effects is often limited by short follow‐up times and lack of detailed clinical observations. There is *weak evidence in favour* of delayed injection site reactions to intra‐muscular injections of diminazene aceturate (3.5 mg/kg) that range from mild to severe. There is *weak evidence in favour* of delayed non‐painful but long‐lasting injection site reactions to quinapyramine sulphate/quinapyramine chloride (2.5/1.7 mg/kg s.c.).

## 4 DISCUSSION

Equine trypanosomiasis was defined as a priority equine disease for investing in research, advocacy and improving surveillance in 2015.^2^ The findings of this systematic review support this statement but the general level of evidence provided for the management of equine trypanosomiasis was found to be low with a significant risk of bias and marked heterogeneity. This confirms the hypothesis of this study that objective analysis would help strengthen understanding of current approaches to disease management but that factors relating to study quality and gaps in available knowledge would lead to challenges in having high confidence in conclusions. The included studies are largely descriptive without controls and often contain limited data (none provide an original data set). Diagnostics, follow‐up and clinical acumen were also highly variable thereby limiting any ability to synthesise data.

Causal relationships of interventions could not be inferred from the majority of included manuscripts. Confirming disease resolution is challenging since the disease course can be prolonged and chronic. Equally, a recurrence of clinical signs cannot be determined definitively in the field to be a relapse or a new infection. Collectively, however these reviewed manuscripts do provide some insight into the current situation, challenges and areas of intervention which may have the greatest impact in the treatment and management of equine trypanosomiasis.

### 4.1 Trypanocidal treatment of haemolymphatic disease

Current trypanocidal drugs utilised in equines have value in resolving blood parasitaemia and providing amelioration of clinical signs. Variable follow‐up and uncontrolled studies are a barrier to assessing or comparing the effect size of these available treatments. *Quantifying effect size and optimising treatment regimens* is a priority given the high morbidity and mortality reported here in these treated equines. One study provided an example of how field trials can be performed without complex logistics to provide more valuable data. In Gambia, a randomised non‐inferiority trial compared the use of three trypanocides in field cases with a two week follow‐up, with detailed clinical, clinicopathological and diagnostic data described.^4^

### 4.2 Trypanocidal treatment of neuro‐invasive disease

Given the presented evidence of a guarded prognosis when neurological signs are evident there is a strong case in favour of clinical (and, where permitting, diagnostic) staging of suspected infections with neuro‐invasive *Trypanosoma* sp. This would permit targeted treatment of those equines presenting haemolymphatic clinical signs only where prognosis may be better. There is *weak evidence in favour* of exploring curative treatment in *T. equiperdum* infections/exposure where neuropathology is not noted. It is possible that the described protocol is novel in timing and intensity of treatment rather than the particular trypanocides selected. In the absence of more data, a similar approach could logically be applied to all neuro‐invasive *Trypanosoma* sp. if the time of exposure was known.

To implement a strategy which aims to treat cases before neuro‐invasion, early intervention would be important (although case evolution does appear variable). Auty et al.^41^ noted that administration of a trypanocide within two days of the onset of clinical signs (facilitated by monitoring rectal temperature twice daily) resulted in a rapid recovery. Delayed treatment was associated with a prolonged recovery and signs of chronic disease (ataxia, anaemia, weight loss, behaviour changes) which were risk factors in case fatality.^41^ This aligns with a priori knowledge from laboratory models in mice^44^ and treatment protocols for Human African Trypanosomiasis.^45^ Implementation of early treatment is likely to be a challenge. In the analysed studies, duration of clinical signs reported prior to presentation was often weeks or months and reports of antibiotics as first‐line treatment were not uncommon. *Whilst this analysis does point to a need for more effective trypanocidal drugs, collectively these manuscripts also illustrate a more pressing requirement to strengthen animal health systems*.

There is *weak evidence against* using current trypanocidal drugs for neuro‐invasive *Trypanosoma* sp. where there is evidence of neuropathology. There are no studies in equines that demonstrate a causal positive effect of trypanocidal treatment. This is consistent with the known pharmacological properties of current trypanocides and cross‐species knowledge.^5^ As a clinician, considerations should include the welfare compromise of severe disease to the equine, the reservoir potential of a chronically infected equine, the contribution to the development of trypanocidal resistance and the cost to the owner of treatment and keeping the equine when prognosis is guarded.

### 4.3 Population‐level disease control strategies

Reliance on trypanocidal treatment alone is common (*strong evidence*, *in favour*). The collective evidence here also suggests an absence of coordinated strategic and targeted use of trypanocides for equines and other susceptible and reservoir species. No comprehension is gained from this analysis of the number of equines worldwide receiving regular trypanocidal prophylaxis or treatment. There is one reference to a region‐level multifaceted control programme. An initial clinical observation and audit^38^ identified *T. evansi* as an increasing problem in Gujranwala, Pakistan. Implementation of a control programme over 10 years correlated with a reduced prevalence of this pathogen in the local equine population. The control programme included a diagnostic and treatment protocol for equines presenting at the clinic, vector control for the surrounding region and management recommendations.^40^ *Reducing dependence on trypanocides as a single disease control measure is critical*.

### 4.4 Side effects

Concerns of side effects in equines to trypanocides are not unfounded but *this analysis suggests they are uncommon*. This is with the caveat that clinicopathological data are lacking and the attention to clinical detail and follow‐up is variable across studies. Reports from more than one source and a strong temporal relationship (immediate reactions) increase confidence in the association between side effects and treatment. Clusters of side effects within single studies raise suspicions of the involvement of external factors such as batch contamination. The severe reaction to intravenous administration of isometamidium noted in three studies is documented in other species. The pain response that can be noted at injection and localising clinical signs (e.g. scratching neck with hindlimb) implies vascular irritation. The systemic reaction has been speculated to be related to a low therapeutic index and dose‐dependent toxicity due to an anticholinesterase effect^46^, ^47^ although the clinical picture described here with profound tachycardia does not support this mechanism. Accurate bodyweight estimation, minimising plasma peak concentration by administering the dose in two aliquots and increasing the time frame for administration (by dilution in a larger volume) are all valid approaches to minimise risk. *No data are presented on side effects that could occur from multiple dosing for prophylaxis or treatment over a number of years* (*for example on organ function*) *which is a key knowledge gap*.

## 5 CONCLUSIONS

In conclusion, this systematic review highlights the challenges in making such progress in the management of a disease that predominates in lower and middle‐income countries.

Evaluation of reported current disease management strategies for equine trypanosomiasis highlighted the over‐reliance upon trypanocides as a single measure together with a lack of evidence evaluating treatment efficacy. A greater focus on multi‐faceted, multi‐species disease control is required incorporating other species, in particular cattle that are important in the disease epidemiology. The consideration of working equines in livestock trypanosomiasis management strategies (where relevant) should be a natural progression from their inclusion in the Terrestrial Animal Health Code.^48^

This compilation emphasises areas of missing knowledge. There is a clear data bias towards trypanosomiasis reporting in horses despite mules and donkeys forming the majority proportion of the global equine population.^49^ This may reflect the economic interest of horses or be a true reflection of a lesser disease burden within donkeys and mules. Going forward, greater consideration to mules and donkeys is warranted including exploration of species‐specific pharmacokinetics of trypanocides.

Experimental design is a key area of weakness; the majority of reported manuscripts are descriptive, observational studies without controls or randomisation and it is often challenging to extract data. There are no available open data sets. Open data sets and the use of causal inference analysis could allow utilisation of observational clinical data most effectively. Greater consideration of priority research questions, optimising study design to incorporate both restrictions that may be placed by resources and time, standardisation of practical diagnostic protocols (to facilitate comparison) and capacity building of clinical acumen and field‐based research skills of local vets and scientists could improve the quality of collected data and optimise the use of available resources. Priority topics for well‐planned field trials include evaluating optimal regional disease management regimens, including the effect sizes of the individual interventions and a consideration of the value of treating neurotrypanosomiasis with current therapeutics, taking into account welfare implications, reduced productivity and cost involved to the owner.

Data‐driven advocacy is key to accessing funds and it is hoped this systematic review and associated resources can be utilised in developing a strategic, collaborative approach to future work. Many of the concerns highlighted are not unique to trypanosomiasis or to equines but reflect an urgent need to invest in and strengthen animal health systems globally.^50^

This systematic review of the management of disease caused by equine trypanosomiasis demonstrated that the evidence for effective treatment of equine trypanosomiasis is variable. It successfully highlighted existing knowledge and identified gaps, thereby revealing potential areas where future challenge‐led research is required.

## FUNDING INFORMATION

Alexandra Raftery was funded by the Vet Fund (University of Glasgow).

## CONFLICT OF INTEREST STATEMENT

The authors declare no conflicts of interest.

## AUTHOR CONTRIBUTIONS

**Alexandra G. Raftery:** Conceptualization; investigation; writing – original draft; methodology; visualization; writing – review and editing; formal analysis; project administration; data curation. **Lauren Gummery:** Writing – review and editing; conceptualization; formal analysis. **Karelhia Garcia:** Conceptualization; writing – review and editing. **Dinesh Mohite:** Conceptualization; writing – review and editing. **Paul Capewell:** Writing – original draft; writing – review and editing; supervision. **David Sutton:** Conceptualization; writing – original draft; writing – review and editing; supervision; data curation.

## DATA INTEGRITY STATEMENT

A Raftery had full access to all the data in the study and takes responsibility for the integrity of the data and the accuracy of the data analysis.

## Supporting information


**Data S1:** Study protocol.

**Figure S1:** Prisma flow diagram.

**Table S1:** Case definition.

**Table S2:** Results of search strategy following removal of duplicates, application of general inclusion and exclusion criteria.

**Table S3:** Extracted data on disease management.

**Table S4:** Risk of bias assessment using ROBINS‐I.

## Data Availability

The data that support the findings of this study are openly available in Figshare at https://doi.org/10.6084/m9.figshare.30803006.
