# Supplementary material for: Equine trypanosomiasis, a systematic review: Disease management
Source: Equine Vet J. 2025 Dec 22;58(2):320–32. doi: 10.1002/evj.70136 (PMC12892392; doi:10.1002/evj.70136)
Supplement: Supplementary file 1 — Data S1: Study protocol. [file EVJ-58-320-s005.pdf]

Data S1:
Equine Trypanosomiasis: A systematic review of the global impact of a neglected tropical veterinary disease: Study protocol

Data repository
All data will be available as supplementary data including raw data, R script and summarised data.

Amendments
The original protocol was uploaded to the Open Science Framework, post-hoc amendments to this protocol are evident from comparison of the two documents.

Funding
Alexandra Raftery is funded by the Vet Fund (University of Glasgow) but was not funded specifically for this study. The authors declare no competing interests.

General methods

Search strategy
Publication year was restricted to between 1980 and 2022 to increase the likelihood that studies would use molecular diagnostics and improve the relevance of the data to current times balanced with aiming to represent data from the maximum number of countries as published surveillance was anticipated to be sporadic.

Source: CAB International: CAB Abstracts and Global Health

Interface/URL: https://www.cabdirect.org/cabdirect/search/?searchtype=advance-search

Database coverage dates: 1930-2022

Search date: July 2022

Search strategy:

((("equine") OR ("equid*") OR ("horse") OR ("donkey") OR ("mule")) AND (("trypanosom*") OR ("surra") OR ("nagana") OR ("dourine") OR ("vivax") OR ("congolonse") OR ("brucei") OR ("equiperdum")))

All search terms searched in ‘All fields’.

Document types: ‘All’

Your products: ‘All products’

Publication year: 1980- present

Source: CAB VetMed Resource

Interface/URL: https://www.cabi.org/vetmedresource/

Database coverage dates: 1912-2022

Search date: July 2022

Retrieved records:

Search strategy:

((("equine") OR ("equid*") OR ("horse") OR ("donkey") OR ("mule")) AND (("trypanosom*") OR ("surra") OR ("nagana") OR ("dourine") OR ("vivax") OR ("congolonse") OR ("brucei") OR ("equiperdum") OR ("evansi") OR ("mal de caderas"))) AND yr:[1980 TO 2020]

All search terms searched in ‘All fields’.

Document types: ‘Abstract’

Source: PUBMED

Interface/URL: https://pubmed.ncbi.nlm.nih.gov

Database coverage dates: 1809-2022

Search Date: July 2022

Retrieved records:

Search strategy:

Search #1: ("Trypanosomiasis, African"[Mesh] OR "Trypanosomiasis"[Mesh] OR "Trypanosomiasis/veterinary"[Mesh] OR "Trypanosomiasis, African/veterinary"[Mesh] OR "Trypanosoma congolense"[Mesh] OR "Trypanosoma vivax"[Mesh] OR "Trypanosoma brucei brucei"[Mesh]) AND ("Horse Diseases"[Mesh] OR "Horses"[Mesh] OR "Equidae"[Mesh])

Year filter: 01/01/1980- PRESENT

Search #2: ("horses"[MeSH Terms] OR "horses"[All Fields] OR "equine"[All Fields]) OR ("equidae"[MeSH Terms] OR "equidae"[All Fields]) OR equid[All Fields] OR ("horses"[MeSH Terms] OR "horses"[All Fields] OR "horse"[All Fields] OR "equidae"[MeSH Terms] OR "equidae"[All Fields]) OR ("equidae"[MeSH Terms] OR "equidae"[All Fields] OR "donkey"[All Fields]) OR ("equidae"[MeSH Terms] OR "equidae"[All Fields] OR "mule"[All Fields])

Year filter: 01/01/1980- PRESENT

Search #3: ("trypanosomiasis"[MeSH Terms] OR "trypanosomiasis"[All Fields]) OR ("trypanosomiasis"[MeSH Terms] OR "trypanosomiasis"[All Fields] OR "trypanosomosis"[All Fields]) OR ("trypanosoma"[MeSH Terms] OR "trypanosoma"[All Fields]) OR congolense[All Fields] OR ("trypanosoma vivax"[MeSH Terms] OR "trypanosoma"[All Fields] OR "vivax"[All Fields]) OR "trypanosoma vivax"[All Fields]) OR brucei[All Fields] OR evansi[All Fields] OR equiperdum[All Fields] OR surra[All Fields] OR "trypanosomiasis, african"[MeSH Terms] OR "african animal trypanosomiasis"[All Fields] OR "nagana"[All Fields] OR "dourine"[All Fields] OR ("mal de caderas"[All Fields])

Year filter: 01/01/1980- PRESENT

Search #4: #2 AND #3

Search #5: #1 OR #4

("trypanosomiasis"[MeSH Terms] OR "trypanosomiasis"[All Fields]) OR "trypanosomes"[All Fields] OR "trypanosomoses"[All Fields] OR "Surra"[All Fields] OR "Nagana"[All Fields] OR "brucei"[All fields] OR "congolense"[All fields] OR "vivax"[All fields] AND ("horses"[MeSH Terms] OR "horses"[All Fields] OR "equine"[All Fields] OR "donkey"[All Fields] OR "ass"[All Fields] OR "mule"[All Fields] OR "equidae"[All Fields])

Source: Web of Science

Interface/URL: https://apps.webofknowledge.com/

Database coverage dates: 1864-2022

Search Date: July 2022

Retrieved records:

Search strategy:

Search #1:

TS= (horse OR equine OR donkey OR ass OR equid* OR mule) OR

TI= (horse OR equine OR donkey OR ass OR equid* OR mule)

Language= Auto

Timespan = 1980-2022

Databases= WOS, BCI, BIOSIS, CABI, CCC, DRCI, DIIDW, KJD, MEDLINE, RSCI, SCIELO, ZOOREC

Search #2

TS= (trypanosom* OR Surra OR Nagana OR brucei OR congolense OR vivax OR evansi OR equiperdum OR mal de cadaras OR dourine) OR

TI= (trypanosom* OR Surra OR Nagana OR brucei OR congolense OR vivax OR evansi OR equiperdum OR mal de caderas OR dourine)

Language= Auto

Timespan = 1980-2022

Databases= WOS, BCI, BIOSIS, CABI, CCC, DRCI, DIIDW, KJD, MEDLINE, RSCI, SCIELO, ZOOREC

Search #1 AND #2

Language= Auto

Timespan = 1980-2022

Databases= WOS, BCI, BIOSIS, CABI, CCC, DRCI, DIIDW, KJD, MEDLINE, RSCI, SCIELO, ZOOREC

Source: Embase: Ovid

Embase 1947-Present, updated daily

Health and Psychosocial Instruments 1985-July 2022

Journals@Ovid Full Text July 07, 2022

Access University of Glasgow Full Text Journals

Books@Ovid July 06, 2022

Ovid MEDLINE® and Epub Ahead of Print, In-Process & Other Non-Indexed Citations, Daily and Versions® 1946 to Present

Interface/URL: https://ovidsp.ovid.com

Database coverage dates: 1946-2022

Search Date:

Retrieved records:

Search strategy:

Search #1:

(equus or equid or equine or horse or donkey or ass or mule or equidae).af.

Timespan: 1980-2022

Search #2:

(trypanosom* or brucei or congolense or vivax or nagana or surra or dourine or evansi or equiperdum or mal de caderas or african animal trypanosomiasis).af.

Timespan: 1980-2022

Search #3: #1 and #2

N.B. af.= all fields

Remove duplicates

Source: Zetoc

Interface/URL: http://zetoc.jisc.ac.uk/

Database coverage dates: 1993-2022

Search Date: July 2022

Search strategy: equine trypanosomiasis

Source: Scopus

Interface/URL: http://www.scopus.com

Database coverage dates: 1904-2022

Search Date: July 2022

Timespan: 1980-2022

Search strategy:

TITLE-ABS-KEY ((horse OR donkey OR mule OR equine OR equus OR equidae OR equid OR ass) AND (trypanosomiasis OR trypanosoma OR trypanosomoses OR brucei OR congolense OR vivax OR surra OR nagana OR dourine OR equiperdum OR evansi OR "mal de caderas"))

Grey literature

Source: Open Access Theses and Dissertations

Interface/URL: http://www.oatd.org

Database coverage dates: 1990-2022

Search Date: July 2022

Search strategy:

((((((trypanosomes OR trypanosomiasis OR trypanosomoses) AND (equine OR equid OR mule OR horse OR donkey))))))

Written in: Any Language

Published in: Any Country

Source: NDLTD

Interface/URL: http://search.ndltd.org

Database coverage dates: 1990-2022

Search Date: July 2022

Search strategy:

"equine" AND "trypanosoma"

Source: ProQuest

Interface/URL: https://search.proquest.com/

Database coverage dates: **-2022

Search Date: July 2022

Search strategy:

(ab(equine) OR ab(horse) OR ab(donkey)) AND ab(trypanosomiasis)

Source: EThOS British Library e-theses online service

Interface/URL: https://ethos.bl.uk/Home.do?new=1

Database coverage dates: **-2022

Search Date: July 2022

Search strategy:

equine OR horse OR donkey OR mule AND trypanosom*

Any word

Source: Research Gate

Interface/URL: www.researchgate.net

Search Date: July 2022

Search strategy:

Equine trypanosomiasis

Donkey trypanosomiasis

Horse trypanosomiasis

Mule trypanosomiasis

Source: WOAH

Interface/URL: https://wahis.woah.org/#/home

Database coverage dates: 2005-2022

Search Date: December 2022

Search strategy: The search tool 'WAHIS' [1] was used to search for outbreaks of or disease status declarations by countries for *Trypanosoma evansi, T. equiperdum* and Tsetse Transmitted Trypanosomiasis.

Study selection and criteria

Abstract review

The abstracts and titles were downloaded and collated into Excel (.csv or .xls). The records were adapted into a standardised format to allow consolidation (Table S2). This allowed searching for duplicity and removal of items listed more than once.

Each title (initial screening for topic relevance) and then title and abstract (abstract review) were reviewed independently by two reviewers who were blinded to the decision of the other reviewer. The articles were selected based upon a predetermined, standardised inclusion and exclusion criteria. Where reviewers differed in opinion, a third reviewer had the deciding vote.

Inclusion criteria:

1. Contained original data on horse, donkey or mule *Trypanosoma* spp. Infection from any country in the world
2. Data complied with case definition (Table S1). Case definition was agreed by AGR, LG and DGMS.
3. All types of study design including case reports were retained to try and obtain the greatest geographical coverage of information since data were anticipated to be sparse/absent in some areas. It was anticipated that the level of evidence would be generally low since RCTs are rare in veterinary medicine and therefore all types of study designed were maintained to gain the benefits of repeated observations.
4. Abstract written in English, French or Spanish due to skillset available to the authors.

Exclusion criteria:

1. Studies reporting experimental infections were excluded.
2. Studies with insufficient information to assess case definition were excluded.
3. Studies where full text was not available were excluded.
4. Non-systematic review articles, book chapters, newspaper articles and other documents that did not contain original data were not included but the bibliographies were reviewed to ensure relevant references had been reviewed for suitability for inclusion.
5. Conference abstracts or theses with peer reviewed manuscripts containing the same data were excluded.

Full text review

Articles meeting these criteria then underwent a review of the full text. This included particular attention that the case definition (Table S1) was still fulfilled when the full text had been reviewed. The included articles were collated into Zotero reference manager (Zotero 5.0.94; https://www.zotero.org/support/credits_and_acknowledgments) to facilitate the review process. Articles requiring full text review in French or Spanish were translated. Articles were excluded after full text review based on the following exclusion criteria. This review was performed by two observers with a third as the decision maker for differing opinions.

Exclusion criteria for full text:

1. Study did not reach criteria (inclusion/exclusion) outlined in abstract review.

Data extraction and management

For included manuscripts, data was extracted and entered into the Excel spreadsheet. Inclusion and exclusion criteria were determined for each of the five study questions. For all studies the following information was recorded:

General information

Primary author, research team (from studied country, from abroad, collaboration), manuscript title, year of publication, country studied, language of manuscript, funding (internal, external collaboration), DOI, study design (Case report/Case series/Cohort study/Longitudinal study/Case control study/Randomised controlled trial/Meta-analysis/Systematic review; Observational/Interventional; Retrospective/Prospective), exploratory or confirmatory study, hypothesis stated?, results confirm (negative result) or reject null hypothesis (positive result).

Extraction of specific data

For each of the three questions specific inclusion and exclusion criteria were generated to select relevant data within the generated dataset. Papers could be included in more than one question and there were some data that was utilised for multiple questions.

1. What is the global geographical distribution and prevalence of equine trypanosomiasis? In equines residing in low- and-middle income countries is trypanosomiasis more prevalent than those in higher income countries?

Inclusion criteria:
Reports a population of equines (donkey, horse or mule) screened for *Trypanosoma* spp. using any diagnostic method.

Exclusion criteria
Studies were excluded if the number of equines positive or negative by the diagnostic method could not be extracted

Variables recorded
N.B. The WAHIS tool [1] was used as an additional source of information for outbreak information.

Number of equines, outbreak/surveillance/clinic, diagnostic method (clinical signs, microscopy, molecular, serology), number positive for 1 or more *Trypanosoma* spp., number positive for *T. congolense*, number positive for *T. vivax*, number positive for *T. brucei* sp. (*T. b. evansi/T. b. equiperum/T. b. brucei/T. b.* g*ambiense/T. b. rhodesiense*/Not known), percentage positive.

The most current data available from the FAO [2] was extracted on equine populations for every country in the world (n=193). Data on the income status (high income or lower/middle income country) of each country was imported. [3]

Data analysis
Outcome variable: The number positive for 1 or more *Trypanosoma* spp. and the total number of tested animals were used. A distinction was made between outbreak data and surveillance/clinic data. Where multiple studies were available for one country the data were collated. The estimated point prevalence and 95% confidence intervals were calculated for each country where data were available. The country level risk for infection with trypanosomiasis were categorised countries (Table S3). The proportion of high-income countries reporting equine trypanosomiasis was compared to the proportion of low- or middle-income countries reporting trypanosomiasis.

Data visualisation
The number of equines at high, medium, low and no/unknown risk from equid trypanosomiasis were crudely estimated using the data on equine populations from FAO [2] and presented visually using Excel. The species of *Trypanosoma* spp. reported in each country were also presented visually using Excel.

Anticipated limitations

i. Small sample sizes, infrequent surveying and small areas of geographical representation of screened populations.
ii. Bias from selection criteria in studies and publication bias (lack of reporting of negative results).
iii. A large degree of heterogeneity was expected due to variation of sensitivity and specificity of diagnostic methods and in prevalence of disease (impacting upon the positive and negative predictive values for the tests).
iv. Surveillance data may not be published, countries with no known disease burden are unlikely to test (except for during import testing), LMIC countries may not have the resources to run surveillance programs.

2. In equines, is trypanosomiasis infection a significant contributor to global morbidity and mortality?

Inclusion criteria

1. Study describes number of animals with specific clinical signs of trypanosomiasis in a screened population or outbreak
2. Cases are confirmed with appropriate diagnostic test (per case definition)

Variables recorded
Number of equines, number of confirmed cases, number of symptomatic confirmed, outcome variable 1 (morbidity)- pyrexia (>37.8°C donkey, >38.5°C horse), anaemia(<26% donkey, <30% horse), [4,5] poor body condition (<2/5 [6]), oedema, abortion, number with haemolymphatic disease, number with

neurological signs (myelitis/encephalitis, peripheral neuropathy; outcome variable 2 (mortality)- number of fatalities.

Data analysis
Descriptive information of included studies.

Outcome variable: The percentage of confirmed equine cases of trypanosomiasis with documented morbidity (haemolymphatic and/or neurological disease) and mortality was calculated. The death to case ratio was calculated. These outcomes were summarised (median +/- IQR) for included studies.

Data visualisation
Descriptive statistics for included studies summarised in table format focused upon comparison of outcome variables.

Study	Study design	Infection rate	Morbidity	Mortality and death to case ratio	Asymptomatic animals?	Timeframe & follow-up	GRADE

Anticipated limitations

i. Sources of heterogeneity e.g. studies using insensitive diagnostics such as microscopy will lead to diagnoses only in highly parasitaemic animals.

ii. Small sample sizes.

iii. Few studies with detailed recording of clinical signs and variable veterinary skill levels between countries.

iv. If longitudinal data is not available frequency of progression of disease towards the most severe phenotype of neurological disease or death cannot be assessed.

3. In equines, what are the effects of disease management strategies of trypanosomiasis on disease severity (individual level) and disease prevalence (population level) compared to no intervention?

Inclusion criteria

i. Interventional study examining a disease management strategy (trypanocidal treatment, vector control, vector repellent, proximity to reservoir species).

ii. Number of equines tested positive in sampled equine population examined before and after intervention.

Variables recorded
Number of equines, intervention studied, presence of control group?, *Trypanosoma* spp tested for, diagnostic method, timeframe for repeat evaluation, outcome variable, percentage positive before intervention, percentage positive after intervention, percentage change associated with intervention, side effects (to trypanocides).

Data analysis
Descriptive only focussing upon outcome variable(s) measured (i.e. clinical improvement or change in diagnostic test result positivity in response to intervention) and qualitative heterogeneity assessment.

Data visualisation
Tables: Descriptive statistics of included studies by intervention (assessed by disease severity (individual) or disease prevalence (population) summarised in table format focused on comparison of outcome variables, subcategorised by *Trypanosoma* spp..

Intervention	Study design	Baseline	Post intervention

Anticipated limitations

i. Few interventional studies performed

ii. Lack of control groups

iii. Outcome variables will vary

iv. Timeframe for repeat evaluation will vary

Critical assessment of methodological quality

For each of the three questions, the level of confidence in individual studies were assessed using GRADE [7] to rate certainty as very low, low, moderate or high. Certainty was rated down for risk of bias, imprecision, inconsistency, indirectness and publication bias and certainty was rated up for a large magnitude of effect, dose-response gradient and residual confounding factors that would increase effect. Question level outcomes were described as strong or weak evidence, in favour or against.

Risk of bias assessment

It was anticipated that quality of studies was likely to be mixed and generally low therefore bias assessment may have to be more subjective. However where possible the risk of bias for each included study was assessed by utilising the ROBINS-1 tool [8] for non-randomised trials. *Robvis* [9] was used to visualise the risk of bias assessment. An overall risk of bias judgement for each specific outcome (study questions 1-3) was categorised as low, high or some concerns. [10]

Demographics of included studies

A flow chart demonstrating the number of included studies was made using the PRISMA template. [11] Descriptive statistics of the study designs, publication year, sex of primary author and whether the affiliation of the primary author was from the country that the data was collected were presented. The number of studies reporting positive results was calculated.

Sources of funding for reported studies

Sources of funding where reported were recorded for included studies (Table S2).

Data analysis

A qualitative assessment of heterogeneity was made and limitations recognised prior to performing 'Synthesis without meta-analysis'. [12] Where indicated data were synthesised utilising meta-analysis. Meta-analyses using extracted proportion data were performed using an inverse variance method with a random effects model (with Hartung-Knapp adjustment) and logit transformation. For individual studies, Clopper-Pearson confidence intervals were calculated. τ2 (using a DerSimonian-Laird estimator) and I2 (based on Q) were calculated as quantitative measures of heterogeneity. The results were visualised in forest plots. Analyses were performed using 'metafor' and 'meta' in R studio. R script is available in Data S2 of Part 1 of this study.[14]

Justification for any deviations from the protocol

The original protocol can be accessed with the pre-registered documents so post-hoc analysis and/or changes are clear.

References

1. WAHIS. https://wahis.woah.org/#/dashboards/country-or-disease-dashboard. Accessed 18 October 2022.

2. FAOSTAT, F. Live Animals. http://faostat.fao.org/beta/en/#data/QA. Accessed 31 October 2016.

3. DAC List of ODA Recipients - OECD. https://www.oecd.org/dac/financing-sustainable-development/development-finance-standards/daclist.htm. Accessed 23 May 2022.

4. Burden, F.A., Hazell-Smith, E., Mulugeta, G., Patrick, V., Trawford, R. and Brooks Brownlie, H.W. (2016) Reference intervals for biochemical and haematological parameters in mature domestic donkeys (Equus asinus) in the UK. *Equine Vet. Educ.* **28**, 134–139.

5. Clinicopathological Reference Ranges Adult Horses, Rossdales Laboratory. *Rossdales.* https://www.rossdales.com/laboratories/reference-ranges/. Accessed 25 April 2017.

6. Carroll, C.L. and Huntington, P.J. (1988) Body condition scoring and weight estimation of horses. *Equine Vet. J.* **20**, 41–45.

7. What is GRADE? | BMJ Best Practice. https://bestpractice.bmj.com/info/toolkit/learn-ebm/what-is-grade/. Accessed 25 June 2020.

8. Sterne, J.A., Hernán, M.A., Reeves, B.C., Savović, J., Berkman, N.D., Viswanathan, M., Henry, D., Altman, D.G., Ansari, M.T., Boutron, I., Carpenter, J.R., Chan, A.-W., Churchill, R., Deeks, J.J., Hróbjartsson, A., Kirkham, J., Jüni, P., Loke, Y.K., Pigott, T.D., Ramsay, C.R., Regidor, D., Rothstein, H.R., Sandhu, L., Santaguida, P.L., Schünemann, H.J., Shea, B., Shrier, I., Tugwell, P., Turner, L., Valentine, J.C., Waddington, H., Waters, E., Wells, G.A., Whiting, P.F. and Higgins, J.P. (2016) ROBINS-I: a tool for assessing risk of bias in non-randomised studies of interventions. *BMJ* **355**. https://www.bmj.com/content/355/bmj.i4919. Accessed 25 June 2020.

9. McGuinness, L.A. and Higgins, J.P.T. Risk-of-bias VISualization (robvis): An R package and Shiny web app for visualizing risk-of-bias assessments. *Res. Synth. Methods* **n/a**. https://onlinelibrary.wiley.com/doi/abs/10.1002/jrsm.1411.

10. Chapter 8: Assessing risk of bias in a randomized trial. /handbook/current/chapter-08. Accessed 25 June 2020.

11. PRISMA. http://prisma-statement.org/PRISMAStatement/FlowDiagram. Accessed 29 June 2020.

12. Campbell, M., McKenzie, J.E., Sowden, A., Katikireddi, S.V., Brennan, S.E., Ellis, S., Hartmann-Boyce, J., Ryan, R., Shepperd, S., Thomas, J., Welch, V. and Thomson, H. (2020) Synthesis without meta-analysis (SWiM) in systematic reviews: reporting guideline. *BMJ* **368**. https://www.bmj.com/content/368/bmj.l6890. Accessed 1 July 2020.

13. Raftery, A.G., Gummery, L., Garcia, K., Mohite, D., Capewell, P. and Sutton, D.G.M. (2025) Equine trypanosomiasis, a systematic review and meta-analyses: Prevalence, morbidity and mortality. *Equine Vet. J.* https://beva.onlinelibrary.wiley.com/doi/10.1111/evj.70101
