# Supplementary material for: Equine trypanosomiasis, a systematic review: Disease management
Source: Equine Vet J. 2025 Dec 22;58(2):320–32. doi: 10.1002/evj.70136 (PMC12892392; doi:10.1002/evj.70136)
Supplement: Supplementary file 2 — Figure S1: Prisma flow diagram. [file EVJ-58-320-s001.pdf]

Equine Veterinary Journal BEVA

Figure S1: PRISMA 2020 flow diagram for new systematic reviews which included searches of databases and registers only.

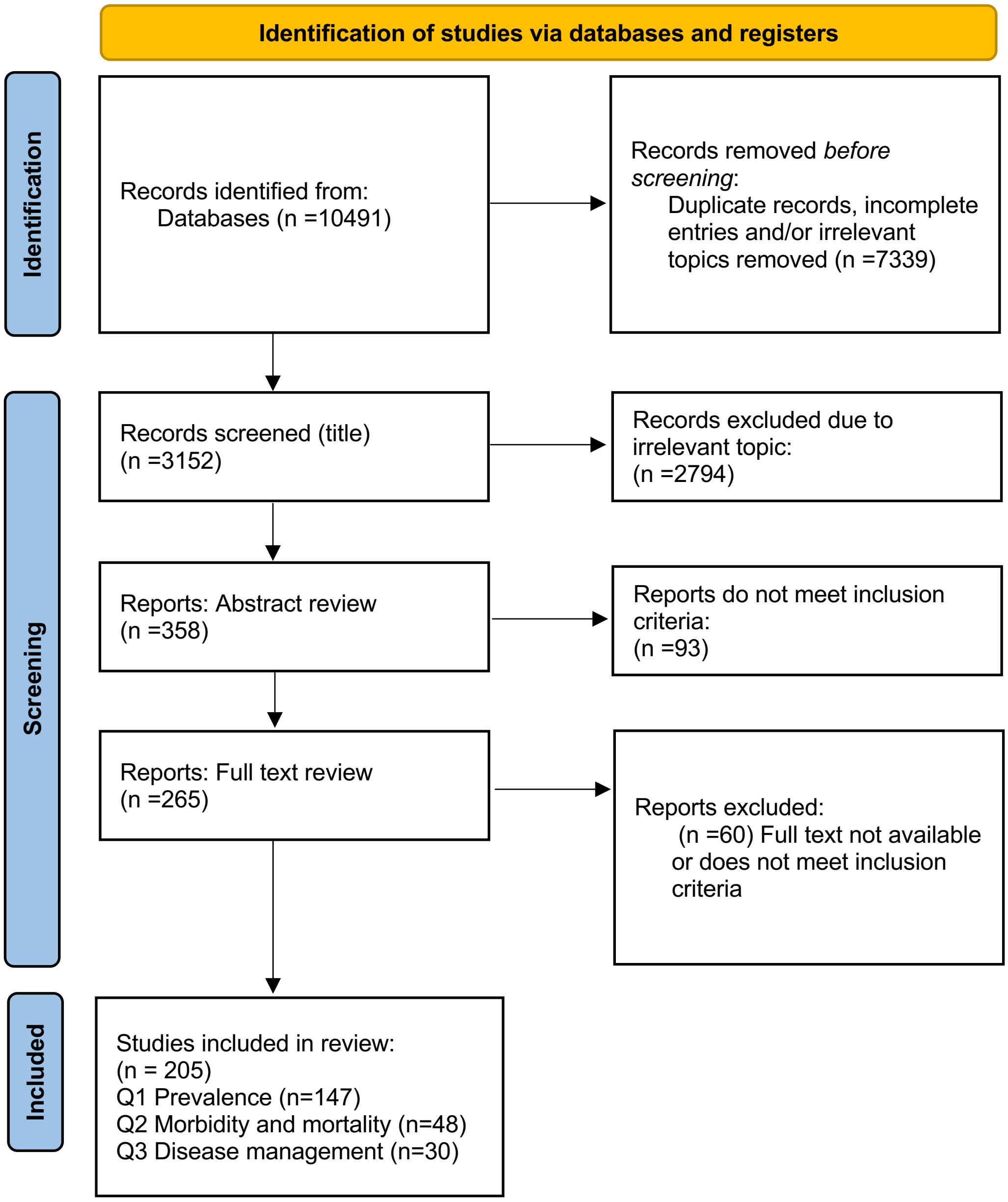

*Consider, if feasible to do so, reporting the number of records identified from each database or register searched (rather than the total number across all databases/registers).

**If automation tools were used, indicate how many records were excluded by a human and how many were excluded by automation tools.

From: Page MJ, McKenzie JE, Bossuyt PM, Boutron I, Hoffmann TC, Mulrow CD, et al. The PRISMA 2020 statement: an updated guideline for reporting systematic reviews. BMJ 2021;372:n71. doi: 10.1136/bmj.n71

For more information, visit: http://www.prisma-statement.org/
