# Supplementary material for: Equine trypanosomiasis, a systematic review: Disease management
Source: Equine Vet J. 2025 Dec 22;58(2):320–32. doi: 10.1002/evj.70136 (PMC12892392; doi:10.1002/evj.70136)
Supplement: Supplementary file 3 — Table S1: Case definition. [file EVJ-58-320-s004.pdf]

Equine Veterinary Journal BEVA

Table S1: Case definition for inclusion of manuscripts.

	**Clinical signs** consistent with trypanosomiasis:	
		Haemolymphatic
	AND/OR	Neuropathological
AND	**Laboratory diagnostic**	
		Molecular: PCR or LAMP
	OR	Antibody: ELISA, LFA, CATT
	OR	Microscopy: Positive identification of *Trypanosoma* sp. on examination of wet blood smear, buffy coat or Giemsa/Diff quik stained blood smear
