# Supplementary material for: Equine trypanosomiasis, a systematic review: Disease management
Source: Equine Vet J. 2025 Dec 22;58(2):320–32. doi: 10.1002/evj.70136 (PMC12892392; doi:10.1002/evj.70136)
Supplement: Supplementary file 6 — Table S4: Risk of bias assessment using ROBINS‐I. [file EVJ-58-320-s006.pdf]

Equine Veterinary Journal BEVA

Table S4: Risk of bias assessment using ROBINS-I.

Study	D1	D2	D3	D4	D5	D6	D7	Overall
Shaikh et al (2016)	!	!	+	+	!	X	X	X
Waheed et al (1998); Waheed et al (2003), Gondal & Ahmad (2010)	!	X	-	-	X	-	X	X
Bhatt et al (2010)	!	!	+	+	!	X	X	X
Bharkad et al (2005)	!	!	+	+	+	X	X	X
Monzon et al (2003)	!	!	+	+	!	-	-	X
Berlin et al (2010)	!	+	+	+	-	-	-	-
Raftery et al (2019)	+	+	+	+	-	+	-	+
Assefa and Abebe (2001)	!	-	+	+	-	-	-	-
Tamarit et al (2010)	!	+	+	+	X	-	-	-
Laha et al (2007)	!	+	+	+	X	X	-	X
Davkharbayar et al (2020)	!	!	+	+	+	X	X	X
Tuntasuvan et al (2003)	!	!	+	+	+	-	-	-
Kihurani et al (1994)	!	!	+	+	X	-	-	X
Auty et al (2008)	!	+	+	-	+	+	-	-
Faye et al (2001)	!	!	+	+	+	X	X	X
Yadav et al (2012)	!	+	+	+	-	-	-	-
Da Silva et al (2011)	!	-	+	+	-	-	-	X
Camoin et al (2011)	!	+	+	+	-	+	+	-
Reck et al (2020)	!	!	+	+	X	X	-	X
Kumar et al (2020)	!	!	+	+	!	X	-	X
Ahmed et al (2010)	!	-	+	+	!	X	+	X
Moraes et al (2007)	!	!	+	+	-	X	X	X
Dehoux et al (1996)	!	!	+	+	!	X	X	X
Saqib et al (1998)	!	!	+	+	!	X	X	X
Silva et al (1995)	!	!	+	+	!	X	!	X
Singh et al (2012)	!	!	+	+	!	X	X	X
Ranjithkumar et al (2014)	!	!	+	+	-	-	-	X
Bhardwaj et al (2007)	!	!	+	+	!	X	X	X
Dedar et al (2014)	!	!	+	+	!	X	X	X
Dhollander et al (2006)	!	X	-	-	!	-	-	X

Domains:
D1: Bias due to confounding.
D2: Bias due to selection of participants.
D3: Bias in classification of interventions.
D4: Bias due to deviations from intended interventions.
D5: Bias due to missing data.
D6: Bias in measurement of outcomes.
D7: Bias in selection of the reported result.

Judgement
! Critical
X Serious
- Moderate
+ Low
